# ImmunoPET in Multiple Myeloma—What? So What? Now What?

**DOI:** 10.3390/cancers12061467

**Published:** 2020-06-04

**Authors:** Clément Bailly, Benjamin Chalopin, Sébastien Gouard, Thomas Carlier, Patricia Remaud-Le Saëc, Séverine Marionneau-Lambot, Philippe Moreau, Cyrille Touzeau, Françoise Kraeber-Bodere, Caroline Bodet-Milin, Michel Chérel

**Affiliations:** 1 CRCINA, INSERM, CNRS, Université d’Angers, Université de Nantes, 44093 Nantes, France; clement.bailly@chu-nantes.fr (C.B.); benjamin.chalopin@yahoo.fr (B.C.); sebastien.gouard@univ-nantes.fr (S.G.); thomas.carlier@chu-nantes.fr (T.C.); patricia.lesaec@univ-nantes.fr (P.R.-L.S.); severine.marionneau-lambot@univ-nantes.fr (S.M.-L.); francoise.bodere@chu-nantes.fr (F.K.-B.); caroline.milin@chu-nantes.fr (C.B.-M.); 2 Nuclear Medicine Department, University Hospital of Nantes, 44093 Nantes, France; 3 Department of Hematology, University Hospital of Nantes, 44093 Nantes, France; philippe.moreau@chu-nantes.fr (P.M.); cyrille.touzeau@chu-nantes.fr (C.T.); 4 Nuclear Medicine Department, ICO-René Gauducheau Cancer Center, 44800 Saint-Herblain, France

**Keywords:** immunoPET, multiple myeloma, immunotherapy, precision imaging, companion imaging agents, heterogeneity, theranostic, daratumumab, PD-1/PD-L1, pharmacokinetic

## Abstract

Despite constant progress over the past three decades, multiple myeloma (MM) is still an incurable disease, and the identification of new biomarkers to better select patients and adapt therapy is more relevant than ever. Recently, the introduction of therapeutic monoclonal antibodies (mAbs) (including direct-targeting mAbs and immune checkpoint inhibitors) appears to have changed the paradigm of MM management, emphasizing the opportunity to cure MM patients through an immunotherapeutic approach. In this context, immuno-positron emission tomography (immunoPET), combining the high sensitivity and resolution of a PET camera with the specificity of a radiolabelled mAb, holds the capability to cement this new treatment paradigm for MM patients. It has the potential to non-invasively monitor the distribution of therapeutic antibodies or directly monitor biomarkers on MM cells, and to allow direct observation of potential changes over time and in response to various therapeutic interventions. Tumor response could, in the future, be anticipated more effectively to provide individualized treatment plans tailored to patients according to their unique imaging signatures. This work explores the important role played by immunotherapeutics in the management of MM, and focuses on some of the challenges for this drug class and the significant interest of companion imaging agents such as immunoPET.

## 1. Introduction

Multiple myeloma (MM) is a haematological malignancy characterised by the clonal proliferation of malignant plasma cells within the bone marrow [1]. It is a rare disease that accounts for approximately 80% of malignant monoclonal gammopathies and 15% of haematological malignancies. This pathology preferentially affects individuals over 40 years of age with a peak frequency between 65 and 70 years of age [2]. Prior to the 1990s, the limited therapeutic arsenal meant that physicians had to wait for the onset of clinical symptoms (formerly known as CRAB criteria) before initiating therapeutic management. Fortunately, the therapeutic landscape for MM has considerably changed since then, thanks in part to the arrival of new molecules, such as proteasome inhibitors, which have changed the prognosis of the disease. In addition, a better understanding of tumour biology has led to a profound update of the diagnostic criteria and therapeutic indications for MM. The disease is no longer defined by symptoms alone, but by a set of biomarkers that take into account clinical, biological, immunological, cytogenetic and imaging data [3]. This radical change in patient management, combining the use of modern therapy and personalized management, has resulted in a significant increase in the overall survival of patients with MM. The median survival of newly diagnosed MM patients has thus increased from about 2.5 years in the 1990s to almost 5.5 years today [2,4,5]. Some patients even show an improvement in recurrence-free survival of more than 10 years [6]. However, despite this progress, MM is still an incurable disease, and the identification of new biomarkers to better select patients with poor prognosis eligible for more intensive therapy is more relevant than ever. Recently, the introduction of therapeutic monoclonal antibodies (mAbs) (including direct-targeting mAbs and immune checkpoint inhibitors) appears to have changed the paradigm of MM management, emphasizing the opportunity to cure MM patients through an immunotherapeutic approach. In this context, immuno-positron emission tomography (immunoPET), combining the high sensitivity and resolution of a PET camera with the specificity of a radiolabelled mAb, holds the capability to cement this new treatment paradigm for MM patients [7]. It has the potential to non-invasively monitor the distribution of therapeutic antibodies or directly monitor biomarkers on MM cells, and to allow direct observation of potential changes over time and in response to various therapeutic interventions. Tumor response could, in the future, be anticipated more effectively to provide individualized treatment plans tailored to patients according to their unique imaging signatures. This work, based on a reflective model by Rolfe et al. [8], explores the important role played by immunotherapeutics in the management of MM, and focuses on some of the lessons and challenges for this drug class and the significant interest of companion imaging agents such as immunoPET.

## 2. What?

What do we need to take immunotherapy in MM to the next level? The history of MM treatment has been punctuated by small revolutions. The treatment landscape of MM has indeed incrementally evolved over the past three decades, resulting in significant improvements in patients’ outcomes [1,2,5]. Until recently, proteasome inhibitors and immunomodulatory drugs were the backbone of the current standards of MM treatment in the front line, in the relapse and maintenance settings [6]. In 2015, the FDA approval of two therapeutic mAbs, elotuzumab and daratumumab, marked the last major milestone in MM treatment and initiated the era of immunotherapy [9]. Notably, after showing extraordinary efficacy in the treatment of relapsed or refractory MM, both as monotherapy and in combination with standard therapies, daratumumab demonstrated tremendous promise in first-line therapy in subjects eligible or not for intensive treatment [10,11,12,13,14]. Readers wishing to have a more complete overview of these trials’ results and combination studies can refer to several excellent papers dealing with this issue [10,15,16]. Moreover, the additive toxicity seems to be limited when combining these two mAbs with conventional MM treatment options in different settings [17,18]. These data have now put these two mAbs and, particularly, daratumumab, into a leading role in the management of MM patients in second-line and first-line therapy.

The main strength of therapeutic mAbs used in MM lies in their ability to recruit innate and adaptive immune cells and in the ubiquitous nature of their targets. This action mechanism differs from standard small-molecule drugs. Elotuzumab targets the extracellular domain of the signalling-lymphocytic-activation-molecule F7 (SLAMF7) [15]. This protein, also referred to as CS1, plays an important role in MM cell adhesion to bone marrow stromal cells. Elotuzumab inhibits this interaction, enhances antibody-dependent cellular cytotoxicity (ADCC) and activates natural killer (NK) cells. For its part, daratumumab targets CD38, a cell surface glycoprotein with ecto-enzymatic and receptor functions [17,19]. The action mechanisms of daratumumab include ADCC, antibody-dependent cellular phagocytosis (ADCP), complement-dependent cytotoxicity (CDC) and also direct apoptosis induction via cross-linking and inhibition of ecto-enzymatic function. In addition, it also improves immunomodulation by the elimination of CD38-positive regulatory T cells, regulatory B cells, and myeloid-derived suppressor cells. Furthermore, by contrast to the complex clonal genomic heterogeneity of MM, mAbs’ targets are regarded as more ubiquitous, which makes this class of agents very attractive for elimination of all MM subclones. Indeed, independently of their cytogenetic and molecular signature, more than 95% of MM patients express SLAMF7 and CD38.

Besides, pharmacokinetic profiles of therapeutic monoclonal antibodies have been extensively described [20,21,22,23,24,25,26]. The latter are characterized by low clearance, limited volume of distribution (consistent with limited distribution out of the vascular space), and low variability. Therapeutic mAbs are catabolized in a large number of tissues and clearance is not limited to a single organ. Therefore, intrinsic factors such as body weight, age, sex or tumour burden are expected to have no significant impact on their exposure. Similarly, as they are unable to cross the glomerular membrane due to their large size, renal impairment has limited effect on them. Finally, the high-affinity interaction of mAbs and their targets explains non-linear elimination, mainly due to target-mediated drug disposition. All of this information has enabled the obtention of theoretical pharmacokinetic models that allow clinically significant concentrations to be maintained [22,23].

Yet, a cloud remains on the horizon. For reasons still under investigation, despite a majority of encouraging results, significant disparities in response quality and duration were observed among MM patients [15,27,28] with direct-targeting mAbs. In reality, approximately 40% of patients show no or limited response (primary resistances) to daratumumab, while, ultimately, all patients undergo relapse or progression secondly despite initial response (secondary resistance).

Similarly, mAbs targeting immune checkpoints and, more specifically, the programmed cell death 1 (PD-1) and PD-1 ligand (PD-L1) pathway have not fully lived up to their expectations [29,30]. Indeed, preclinical data suggest the possible role of the PD-1/PD-L1 axis in immune escape in MM [31]. High PD-L1 concentrations in patients with smoldering MM disease progression or with relapsed and refractory MM supported that its inhibition could be an efficient strategy in this pathology [23,24,25,26]. However, then again, discordant results have been reported [32,33,34]. Early-phase clinical studies reported the lack of activity of single anti-PD-1 pathway agent [35,36] which led to testing a potential synergistic effect of PD-1/PD-L1 blockade in a combined approach with immunomodulatory drugs. Despite better results in phase I trials [37], the FDA brutally suspended the two randomized phase III trials (KEYNOTE-183, KEYNOTE-185) with pembrolizumab associated with lenalidomide and pomalidomide due to high grades of toxicity [38,39]. Based on this observation, many other trials including combined therapies with PD-1/PD-L1 inhibitors have been put on hold.

In consequence, to design the future use of immunotherapy in MM, a few key issues should be addressed. There is a need for a deeper insight into primary and secondary resistance mechanisms, which may involve fluctuating target antigen expression levels on MM cells, as well as into pharmacodynamics endpoints in order to identify biomarkers able to predict response to therapy or to anticipate toxicities. Further, combination therapies or new potential targets for antibody-mediated therapy should be identified and explored. Quoting Caitlin Costello [40], “immunotherapy remains an attractive option for the treatment of multiple myeloma, however, we have much to learn.”

## 3. So What?

Biomarkers allowing the identification of patients who might benefit from therapeutic mAbs are needed. A better insight into disposition profiles resulting in reduced or limited efficacy and into the underlying processes leading to drug resistance is clearly justified. Although these agents have been recently incorporated into the therapeutic arsenal of MM, multiple factors potentially involved in intrinsic and acquired resistance to the latter have been reported [28]: from the senescent phenotype of T cells [41] and the immunogenicity of somatic mutations [42] for immune checkpoint inhibitors, via the baseline number of NK cells [43] and polymorphisms in NK-cell receptors [44] for elotuzumab, to overexpression of complement inhibitory proteins [45] and changes in the CD56 adhesion molecule expression [46] for daratumumab. A precise description of these latter findings is beyond the scope of this short review. This paper focuses on a brief deciphering of factors common to therapeutic antibodies as a class (including direct-targeting mAbs and immune checkpoint inhibitors).

To begin with, low or no target expression could be thought as one of the potential causes of intrinsic resistance, but data in this regard are still under debate. Indeed, several works observed significant correlation between response to daratumumab and basal CD38 expression levels in MM cells [45,47], and similarly with SLAMF7 levels and elotuzumab [43]. However, other authors reported no difference in CD38 expression prior to daratumumab administration between responder and non-responder patients [46,48]. Further studies are thus needed to clarify whether SLAMF7 and CD38 are reliable biomarkers of elotuzumab and daratumumab efficacy. However, the tool used to establish this correlation is equally important. Indeed, following several studies in solid tumours reporting that intratumoral PD-L1 expression prior to treatment provides a higher likelihood of treatment response [49], the FDA approved PD-L1 immunohistochemical assays as complementary diagnostics. Still, in real practice, these tests present many limitations, along with variations among diagnostic sets [49].

In addition, when considering the issue of target expression, it is worth noting that target density and receptor occupancy may also be key factors. Yet, most studies have dealt with receptor occupancy in circulating cells collected from peripheral blood and no data at the lesion level have systematically been made available [26,50,51]. It is generally assumed that the amount of drug reaching the target in the vascular compartment is a good reflection of the tumour microenvironment. Regarding the low tissue-to-blood ratio of most therapeutic mAbs, the extent to which the expected level of exposure in the blood will lead to correlated effects at the tumour level remains to be determined [52]. Making the measurement of receptor occupancy in circulating cells thereby remains a purely speculative pharmacodynamic endpoint. Along the same lines, variability in the pharmacokinetics of mAbs is generally studied through systemic drug exposure [53] and rarely using drug concentrations in the lesions of interest, which take into account tumour accessibility or drug penetration [54,55]. The latter may yet seem complex to achieve in MM, with heterogeneous focal lesions having unique profiles, in different niches of the bone marrow [56,57].

The evaluation and definition of the exposure-response pattern has also been made more difficult by time-dependent variations in pharmacokinetics of these therapeutic mAbs [22,28,51,58]. Although the exact reasons for this time-varying clearance have so far been unclear, it has been speculated that the change in clearance of these antibodies could be due to decreases in tumour load or secondary to a general protein turnover in the body. Therefore, initial weekly dosing of most therapeutic antibodies is thought successful in overcoming the initial high target-mediated clearance and in rapidly establishing effective concentrations. Thereafter, dosing every 2 to 4 weeks is theoretically sufficient to maintain target saturation. However, this does not take into account a potential mechanism of acquired resistance: the modulation of target expression after treatment. Indeed, with daratumumab for example, the release of CD38 from MM cells by microvesicles and the transfer of CD38–daratumumab complexes from myeloma cells to monocytes and granulocytes have been reported [59,60]. Similarly, development of some level of immunogenicity may lead to impaired activity of therapeutic mAbs. Anti-drug antibodies (ADAs) may be associated with reduced biological activity, altered clearance, plasma half-life and tissue distribution of mAbs. Until now, anti-daratumumab antibodies have not been detected. However, 39% of patients treated with elotuzumab as a single agent tested positive for ADAs, resulting in lower serum trough concentrations, yet with no significant clinical impact [61].

In the classical design of oncology drugs, dose selection has been driven by the maximum tolerated dose paradigm, which may not be an adequate and suitable approach for the selection of the optimal dose of immuno-oncologic agent [62,63]. No sound justification exists to suggest that the maximum tolerated dose is the optimal dose. For most trials, identifying the optimal dose, dosing frequency, and length of treatment for a maximum benefit–risk ratio is nearly impracticable. Established dosing regimens are not optimal, and bedside practice may occasionally provide alternative and more effective or better tolerated drug regimens. This is particularly true when the concept of combination therapy is added. Indeed, the individual pharmacodynamic properties of mAbs are specific to the biology of their target antigen but also directly impact the pharmacokinetic characteristics. Binding to the target antigen, internalization, related intracellular protein catabolism and Fc-mediated effects do not only participate in the mechanism of action of mAbs, but may influence their clearance and those of potential combined agents too [52,64]. This underscores the need to evaluate the pharmacokinetics of each therapeutic mAb used for the treatment of MM, but also the need to repeat the process for any new therapeutic combination [65,66]. With this in mind, the expedited timeline on which anti-PD-1 pathway agents have been developed in MM is a perfect example. The large phase III studies were initiated after only small, single-arm phase I monotherapy trials. The evaluation of different combinations and dosing regimens in complementary larger randomized phase II trials would likely have shed more light on the efficiency and safety of combinations of immunologically active drugs [66].

This last observation is, in fact, part of a wider debate on the actual form of innovation in cancer drugs and the setting up of most clinical trials. Currently, an explosive and unacceptable increase in costs accompanies the development and marketing of anti-cancer drugs [67,68,69]. Combined with unrepresentative results in very limited patient cohorts, insignificant clinical benefits, and more than occasionally, concomitant impairment of patients’ quality of life, the actual clinical research paradigm seems to have outlasted its value in its current form [68,70,71,72].

Finally, technical issues in tumour response evaluation also arose with the use of immunotherapy [73]. Immune checkpoint inhibitors lead to new patterns of response [74,75]. Moreover, the goal of oncologic therapy has evolved from eradicating or curing to containing and controlling disease progression. As such, discontinuation of treatment at the first sign of progression might seem inappropriate. In addition, particularly in MM, better detection and quantification of minimal residual disease is needed as the depth of response to therapy appears to be the most important surrogate for survival [76,77,78]. Yet, it is frustrating to note that despite obtaining a complete response with the recommended assessment techniques, most patients relapse, indicating a residual disease below the level of detection. New approaches are warranted.

In this context, immunoPET appears to be an unrivalled tool with the potential to address all the above issues [7,79]. Only imaging offers the ability to non-invasively investigate spatial and temporal tumour heterogeneity due to perpetual clonal remodelling under the pressure of microenvironment and treatments, a point of major importance for responses to targeted therapies. Furthermore, whereas omics approaches give overall snapshots of tumour biology at a particular moment, molecular imaging can extend this picture and reveal modifications in the expression and distribution of tumour biomarkers in the course of time [80]. In the era of immunotherapy, immunoPET incarnates for MM: in the words of van Dongen et al. [79], “a Navigator in monoclonal antibody development and applications”.

## 4. Now What?

Precision medicine intends to provide the right treatment to the right patient at the right dose, at the right time. ImmunoPET perfectly fits in this strategy. It has previously demonstrated its usefulness in precisely mapping lesion profiles at the molecular level, identifying the available target densities, exploring mAbs targeting capabilities and distribution patterns, assessing treatment response, and predicting adverse events [7,79,81,82,83]. Optimal dosing could even be achieved through sequential imaging. It is true, however, that immunoPET is still in its early stages in MM and that only preclinical data or data reported in small patient samples have been published [84,85,86,87,88]. Nevertheless, there is evidence to suggest that, once exploited, this imaging can have a significant impact on the management of MM patients.

As stated above, intra and inter-lesion tumour heterogeneity favours sampling error, particularly for metastatic disease; the expression of the target in one location does not ensure expression in all locations. Multiclonal heterogeneity is still a major issue in the development of effective strategies, and this is especially true in MM. Multi-regional explorations appear to be essential in this pathology, with spatial disparities in clonal architecture and a potentially uneven distribution of high-risk clonal clusters [56,57]. In addition, biopsy does not evaluate the target’s accessibility to therapeutic agents, and target expression is not necessarily correlated with drugs‘ impact on the target. ImmunoPET imaging, if the biodistribution of the targeted tracer mirrors that of the therapeutic agent, overcomes many of these limitations, allowing exploration of target heterogeneity, assessment of target expression and potential accessibility overview across the whole disease burden, to aid clinical decision making [89]. Two perfect examples were recently published, in which therapeutic mAbs targeting the PD-1/PD-L1 pathway were radiolabelled for immunoPET. Bensch et al. reported the use of 89Zirconium-labelled atezolizumab in a cohort of patients with non-small-cell lung carcinoma (NSCLC), bladder cancer and triple negative breast cancer [90]. In this work, PET tumour uptake was better correlated to response rate and clinical responses than immunohistochemistry or RNA sequencing on tumour biopsies. Similar results were reported by Niemeijer et al. using 89Zirconium-labelled nivolumab in NSCLC patients [91]. ImmunoPET uptake correlated with PD-1 expression assessed by immunohistochemistry and, even if the authors pointed out their small sample size, correlated with response to treatment too. Regarding the discordant results of immune checkpoint inhibitors in MM, this approach could directly be applied in MM patients to longitudinally and non-invasively quantify PD-L1 expression in future immunotherapy studies and the potential of combined therapies. Besides, PET uptake in normal tissues can also serve as a surrogate for the evaluation of immune system activation and immune-related adverse events, the magnitude and severity of which prompted the FDA to stop the KEYNOTE-183 and KEYNOTE-185 trials. Based on Bensch et al.’s reports of tracer uptakes at sites of inflammation, immunoPET could also play a role in the early detection of unknown immune-related adverse events [90].

Although there is general agreement that immuno-PET is a promising tool to assess receptor occupancy in tumours and may aid in optimizing the dose of mAbs required for cancer treatment, only few studies specifically explored this perspective. A notable report was made by Dijkers et al. in a feasibility study of 89Zirconium-labelled trastuzumab in patients with metastatic breast cancer [92]. ImmunoPET imaging allowed the visualization of HER2-positive lesions but most importantly confirmed the dose-dependency pharmacokinetics of trastuzumab and the importance of receptor occupancy. Indeed, at low dose levels of injected tracer, low tumour accumulation was observed in patients, naïve to trastuzumab but with HER2 (human epidermal growth factor receptor-2) positive liver metastasis, due to fast hepatic clearance and low blood levels. Varying doses were necessary for proper tumour uptake in patients already receiving trastuzumab as a treatment and in those naïve to this agent, these latter requiring the administration of more mAbs. Similar explorations of receptor-occupancy and target-mediated pharmacokinetics were also realized in the literature with administration of an excess of unlabelled mAbs. In patients with recurrent CD20+ B-cell lymphoma, Muylle et al. compared distributions of 89Zr-labelled rituximab with and without preload [93]. Although this study was conducted in a small group of patients, there were striking differences in the influence of the standard cold rituximab preload due to circulating targets expressed by B-cells which influenced tracer kinetics. In patients with B-cell depletion, the unlabelled rituximab preload negatively influenced the targeting of tumour lesions by causing partial saturation of CD20 receptors on lymphoma cells. In contrast, in patients with normal circulating CD20+ lymphocyte levels, the cold rituximab preload resulted in improved tumour fixation. In a recent study, Menke-van der Houven van Oordt et al. reported the use of 89Zr-labelled GSK2849330 targeting HER3 (human epidermal growth factor receptor-3) [94]. Biodistribution in tumours and normal tissues was measured with the effect of therapeutic doses of unlabelled GSK2849330 mAbs. Modelling of the absorption kinetics of the radioimmunoconjugate disclosed dose-dependent inhibition of the accumulation rate, suggesting HER3 receptor saturation at the highest mAb doses. This illustrates the ability of immunoPET to display the direct distribution of drugs in patient tissues and to non-invasively determine target engagement, thus providing the ability to select the optimal dose for treatment. Since mAbs bind saturably to their target receptors, one could imagine that there is an ideal dose that results in maximum receptor occupancy and maximum therapeutic effect. Increasing doses should not provide any additional therapeutic advantage but may enhance the risk of toxicity.

**Figure 1:**
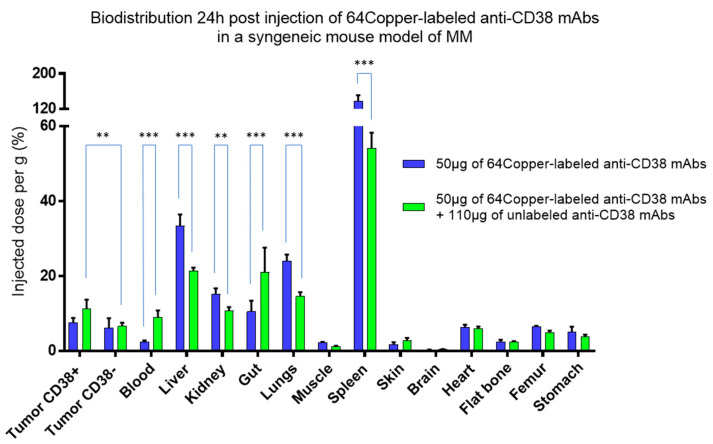
PET-derived biodistribution in a syngeneic model of MM. Tumour-bearing mice were given 50 µg of 64Copper-labelled anti-CD38 mAbs, associated or not to 110 µg of unlabelled mAbs. With only 50 µg of 64Copper-labelled anti-CD38 mAbs, modest accumulation was observed in tumours, with no significant uptake differences between CD38− or CD38+ tumours, due to extensive accumulation of the tracer in the spleen. Adding the unlabelled mAbs dose increases tracer availability over time by “saturating” the spleen, allowing for significant increased CD38+ tumour uptake (non-parametric test). Values are expressed in percentage of the injected radioactive dose per gram of tissue and presented as mean ± SD. **: *p*-value ≤ 0.01; ***: *p*-value ≤ 0.001.

In MM immunoPET, research on optimal dosing, inter-patient variations in pharmacokinetics and tumour targeting is in its infancy. No real clinical cohorts have yet been reported. However, some teams have begun to take an interest in this issue in preclinical studies, including ours [84,85,88,95], and a small first-in-human study was very recently published [88]. Anti-CD38 immunoPET has a high potential in selecting patients and optimal conditions for daratumumab-based treatment. Nevertheless, translating preclinical findings from animal tumour models to clinical results is always challenging. By no means can an animal model reproduce the complexity and heterogeneity of human pathologies. Yet, although used to model only specific aspects, it can rather correctly mimic clinical findings if the chosen model is relevant to the objective. For the purposes of this article, a small experiment was carried out by our team in a syngeneic mouse model of MM. A complete description of the materials and methods can be found in Appendix A. Mice bearing subcutaneous MM tumours were imaged using 64Copper-labelled anti-CD38 mAbs with and without a dose of unlabelled mAbs (Figure 1).

Similar to the clinical findings described by Dijkers et al. [92], the amount of tracer available in the blood and tumour uptakes were influenced by tracer distribution in healthy organs. In this example, the splenic “sink” was responsible for the poor MM CD38+ lesion uptakes. The unlabelled mAbs dose, by “saturating” the spleen, increased tracer availability over time. This model thus perfectly illustrates the dose-dependent kinetics of daratumumab described above and how the preclinical data can fairly closely mirror the clinical data. Indeed, this effect of total mAbs mass and visualization of the splenic “sink” was also briefly discussed in the recent clinical proof-of-principle by Ulaner et al. [88]. AntiCD38 immunoPET could further enhance daratumumab effectiveness. As an aside, this also underlines the importance of using a syngeneic model, a practice unfortunately rare in the literature. Indeed, developed radiotracers are often evaluated in immunodeficient mice with target-expressing human tumour xenografts. This is the case in reported in CD38 studies [84,85,86,88]. Although works by Caserta et al. and Ghai et al. have demonstrated that anti-CD38 imaging is feasible, the relevance of clinical translation is still hampered by the fact that biodistribution and tumour uptake in relation to healthy tissues could not be properly evaluated, tumour xenografts being unrealistically the only site of target expression. This is also illustrated by the necessary co-injection of an excess of isotype control mAbs in Ulaner et al.’s work to avoid anomalous biodistribution of humanized mAbs in highly immunodeficient mice [88,96]. The biodistribution data from our experience in a syngeneic model (Figure 1) may ultimately be closer to what they were able to obtain in patients than their own preclinical model. Human studies with 89Zirconium-labelled and 64Copper-labelled daratumumab immunoPET in MM patients are currently ongoing (trials identifiers respectively NCT03665155 and NCT03311828).

**Figure 2:**
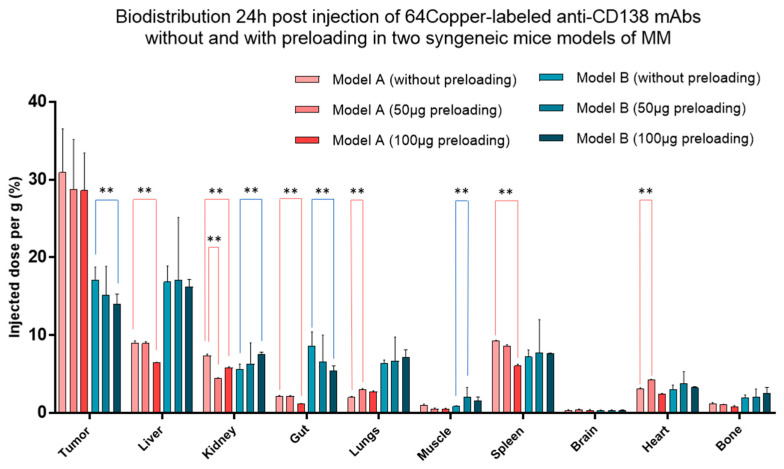
PET-derived biodistribution in two syngeneic models of MM (Model A: MOPC 315 cells in balb/c; Model B: 5T33 MM cells in C57BL/KaLwRij). Tumour-bearing mice were imaged after injection of 64Copper-labelled anti-CD138 mAbs associated or not to 50 µg or 110 µg of unlabelled mAbs 4 h before. Results are completely different from one mouse model to another (non-parametric test). Values are expressed in percentage of the injected radioactive dose per gram of tissue and presented as mean ± SD. **: *p*-value ≤ 0.01.

Besides, preclinical models also represent a valuable tool to explore MM multiclonal heterogeneity. Indeed, as we have seen throughout this paper, the heterogeneity inherent to the distribution of radiopharmaceuticals in target lesions and normal organs suggests that an individualized approach, adapted to the patient, appears essential, particularly from the secondary perspective of the implementation of therapy. Therefore, thinking in portfolio terms, instead of merely switching from one animal model to another, allows the exploration of more complexity and diversity while retaining the strengths of each model [97]. In this context, differences in mAb pharmacokinetics, receptor occupancy or target expression between two models, evaluated with immunoPET, could potentially be seen as simple surrogates of real-world heterogeneity. To illustrate this point, another small experiment was carried out by our team. A complete description of the materials and methods can be found in Appendix B. ImmunoPET using 64Copper-labelled anti-CD138 mAbs was realized in two MM mice models, with and without a dose of unlabelled mAbs (Figure 2).

Similarly to the clinical findings of Muylle et al. [93], the injection of a preload dose led to completely different results, depending on the mouse model. These data suggest weak generalization from one model to another and thus from one MM lesion to another or even from one MM patient to another and the necessity of these titration steps.

Finally, identification of new biomarkers that allow precise measurement of tumour targets for monitoring response to therapy and minimal residual disease assessment are required. These could provide effective tools to rapidly interrupt ineffective treatments and guide other more effective treatment strategies that would benefit patients. Beyond their role as targets for therapy, CD38 and CD138 are among the more interesting ones. Indeed, these two antigens are currently used as standard markers for the identification and purification of MM cells in daily practice. CD38 was previously described. CD138 or syndecan-1 is a cell surface proteoglycan that plays a critical role in the interaction between MM cells and their microenvironment [98]. ImmunoPET targeting these latter thus has the potential to improve MM imaging, especially regarding lesions with low metabolic activity [99].

## 5. What’s More?

ImmunoPET also opens the door to radioimmunotherapy (RIT). Indeed, it provides a useful theranostic companion for targeting assessment and for dosimetric purposes before the administration of therapeutic radioimmunoconjugates [7,79,82]. RIT exploits the immune protein as a carrier to deliver a high dose of therapeutic radiation to tumour cells and limit exposure of normal cells. Since the first reports in the 1980s, RIT has progressively developed [100]. This trend is expected to be boosted by the remarkable success of other theranostic approaches such as radiopeptide or ligand therapy in the treatment of neuroendocrine tumours or prostate cancer. Yet, RIT has also had the opportunity to shine in oncohaematology. In 2004, 90Yttrium-labelled ibritumomab tiuxetan, a mAb targeting CD20, was approved following several clinical studies demonstrating its efficacy in non-Hodgkin B-cell lymphoma (NHL) [101,102,103,104]. In September 2009, it was even approved by the FDA as a first-line therapy for follicular lymphoma patients [105]. However, despite the proven high efficacy in patients resistant to both chemotherapy and rituximab and being undoubtedly the most active treatment regimen ever validated for NHL, this therapeutic approach has never been broadly adopted by the medical community [106,107]. Several reasons have been put forward to explain this failure including the perceived complexity of the delivery and referral process, unfounded economic and logistic considerations and concerns about late radiation toxicity. As these concerns have been dispelled over the last decade, the falling clinical acceptance of anti-CD20 RIT has, above all, demonstrated the critical importance of co-operation among referring physicians, nuclear medicine specialists and industrial partners.

Recently, the increased availability of α-emitter radionuclides, in conjunction with advances in radiochemistry, has led to promising results [108]. Based on their physical characteristics, α-emitters may be particularly effective for MRD treatment, where single cells and small tumour clusters predominate [21]. In this context, our team reported promising preclinical results using antiCD138 mAbs labelled with 213Bismuth and 211Astatine [109,110,111,112]. The potential of RIT and immunoPET in the treatment of lymphomas has not been fully exploited. For the sake of patients, let us not make the same mistake in MM.

## 6. Conclusions

This review provides only a snapshot of the potential value of immunoPET in MM. The principal issue now is the implementation of these imaging approaches in patient care. Indeed, today, the main limitation of immunoPET lies in the challenges that must be met in order to generalize its systematic use, including clinical acceptability and cost. This, of course, also requires for most of these new tracers translation to the clinic, a bumpy road given the complexity of the current regulations for bringing a tracer from bench to actual bedside. The future of immunoPET requires collaboration between PET centres and referring physicians, government authorities that regulate the development of PET probes or fund research, and industrial partners interested in PET imaging or drug development. This is a challenge that deserves to be addressed for the benefits of all MM patients.

## Appendix A

Figure 1 represents the results of a PET-derived biodistribution experiment conducted in a murine syngeneic model of MM. Materials and methods are briefly described below.

Six female C57BL/KalwRij mice were purchased from Envigo and housed under conventional conditions at the Experimental Therapeutic Unit animal facility (SFR François Bonamy, IRS UN, University of Nantes, license number: B-44-278). Experiments were approved by the local veterinary committee (reference 00143.02, 28 March 2014) and carried out in accordance with relevant guidelines and regulations.

Prior to immunoPET, a 5T33-CD38(+) MM cell line was generated by retroviral transduction of the original CD38 negative 5T33 with murine CD38 sequence. Isolated 5T33-CD38(+) clone was then obtained by cloning and screening of the transducted population.

Thirteen days before immunoPET imaging, C57BL/KalwRij mice were grafted subcutaneously on both right and left legs respectively with 2 × 10^6^ 5T33 cells and 2 × 10^6^ 5T33- CD38(+) cells suspended in 100 μL of PBS. Tumours were grown to a size of 0.3–0.8 cm in diameter in line with ethical consideration in animal experiments.

The used antiCD38 mAb was purchased from Biolegend (San Diego, California, CA, USA, ref 102702). Preparation and radiolabelling with 64Copper was carried out following our standard procedure. Briefly, antiCD38 mAb was modified using the copper chelating agent maleimide-DOTA, purified on gel filtration column and radiolabelled as previously described [95,113].

Each mouse was intravenously injected with 50 µg of 64Copper-labelled anti-CD38 mAbs associated or not to 110 µg of unlabelled mAbs. Total activity injected for each mouse was 10 MBq.

ImmunoPET were realized on a multi-modality preclinical imaging system (Inveon™, Siemens Healthcare, Erlangen, Germany). The reconstructed PET images were analysed using Inveon Research Workplace (Siemens Healthcare). Manually drawn 3-dimensional volumes of interest (VOIs) were used to determine tissue uptake values on decay-corrected whole-body coronal images. By assuming a tissue density of 1 g/mL, the VOIs were converted to percentage of the injected radioactive dose per gram of tissue (% ID/g).

Statistical analysis was performed using GraphPad Prism version 7.00. Differences in uptake were tested for significance using the non-parametric Mann–Whitney test for two groups. A *p* value below 0.05 was considered significant.

## Appendix B

Figure 2 represents the results of an experiment in line with several recent works by our team [95,113]. Materials and methods are briefly described below.

Nine female C57BL/KalwRij and nine balb/c mice were purchased from Envigo and housed under conventional conditions at the Experimental Therapeutic Unit animal facility (SFR François Bonamy, IRS UN, University of Nantes, license number: B-44-278). Experiments were approved by the local veterinary committee (reference 00143.01, 28 march 2014) and carried out in accordance with relevant guidelines and regulations.

Twelve days before immunoPET imaging, C57BL/KalwRij and balb/c mice were grafted subcutaneously with, respectively, 2 × 10^6^ 5T33 MM cells and 2 × 10^5^ MOPC 315 (ATCC TIB-23) cells suspended in 100 μL of PBS. Tumours were grown to a size of 0.3–0.8 cm in diameter in line with ethical considerations in animal experiments.

The used antiCD138 mAb was the 9E7.4 mAb, whose characterisation was ensured within our team as previously described [109]. Coupling with TE1PA chelator and radiolabelling with 64Copper was carried out following our standard procedure and as previously reported in recent works [95,113].

Each mouse was intravenously injected with 10 MBq of radiotracer in a volume of 100 μL via the lateral tail vein, associated or not to 50 µg or 100 µg of unlabelled 9E7.4 mAbs, 4 h before.

ImmunoPET and statistical analysis were conducted as previously described in Appendix A.
